# Publication Trends in Aesthetic Breast Surgery: A Bibliometric Analysis

**DOI:** 10.1093/asjof/ojae045

**Published:** 2024-06-10

**Authors:** Roshan Singh Rupra, Kian Daneshi, Dinithi Liyanage, Alessandra Ceccaroni, Antonioenrico Gentile, Ankur Khajuria

## Abstract

**Background:**

Aesthetic breast surgery (ABS) encompasses breast augmentation, breast reduction, mastopexy, and mastopexy augmentation. This topic has seldom been assessed as a bibliometric study. This analysis aims to address this gap and identify trends in ABS literature to guide future research areas. Bibliometrics, the quantitative analysis of publications, particularly scholarly literature, offers valuable insights into research trends and impact.

**Objectives:**

This analysis aims to address this gap and identify trends in ABS literature to guide future research areas.

**Methods:**

The 100 most-cited publications in ABS were identified on Web of Science (Clarivate Analytics, Philadelphia, PA), across all available journal years (from 1953 to 2024). Study details, including the citation count, main content focus, and outcome measures, were extracted and tabulated from each publication. The Oxford Centre for Evidence Based Medicine and levels of evidence (LOE) of each study were assessed.

**Results:**

The 100 most-cited publications in ABS were cited by a total of 11,522 publications. Citations per publication ranged from 46 to 1211 (mean 115.2 ± 135.7), with the highest-cited study being the Pusic BREAST-Q paper (*n* = 1211). A majority of publications were LOE 4 (*n* = 30), representative of the large number of case series. The number of publications for LOE 5, 3, 2, and 1 was 12, 28, 21, and 9, respectively. The main content focus was “outcomes” in 52 publications, followed by “nonoperative management” (*n* = 12) and “surgical technique” (*n* = 12). Patient-reported outcome measures (PROMs) were used in 29 publications, and 53 publications reported aesthetic outcome measures.

**Conclusions:**

This analysis highlights that research methodologies in ABS studies should be improved. This necessary improvement would be facilitated by vigorous, high-quality research, and the implementation of validated ABS-specific PROMs enhancing patient satisfaction, particularly in aesthetic procedures, such as BREAST-Q.

**Level of Evidence: 4:**

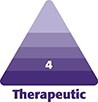

Aesthetic breast surgery (ABS) aims to acquire the ideal breast morphology, and although efforts have been made to quantify this, there is no universal consensus.^1-3^ However, it must be stated that a greater emphasis should also be placed on determining the objective quality of the results, postprocedure. ABS can be broadly subcategorized into: breast augmentation, breast reduction, mastopexy, and mastopexy augmentation.^4^ ABS continues to be one of the most in-demand procedures in plastic surgery.^5^ With techniques advancing, the evolving landscape of patient expectations and aesthetic ideals regarding breast shape and size has been notably influenced by social media platforms, such as Instagram (Menlo Park, CA).^6,7^

Bibliometric studies in healthcare are relatively new but are gaining popularity, being used to analyze large amounts of data and utilize citation counts to identify research trends, including which under-researched areas that need addressing.^8^ It should be noted that although citation count is used to calculate the impact factor of a journal, this is not equivalent to clinical significance.^8,9^

There has been limited bibliometric analysis on ABS.^10^ Our objective is to explore emerging trends in ABS as a whole, identifying current research hotspots. Ultimately, our aim is to provide both surgeons and patients with comprehensive information to enable informed consent. This paper hypothesizes that the top 100 cited articles focused on ABS use predominantly low levels of evidence (LOE) research, with a low use of cosmetic outcome measures—a trend that has been noted in previous bibliometric studies within plastic surgery.^11^

## METHODS

A literature review was performed to identify the 100 most highly cited publications on ABS. All journal publications available on the online database, Web of Science (Clarivate Analytics, Philadelphia, PA), were searched using the following search strategy: “breast augmentation” OR “breast reduction” “mastopexy” OR “patient-reported outcomes” OR “PRO” OR “patient satisfaction” OR “mammaplasty” OR “outcome measures” OR “quality of life” OR “outcome assessment” OR “aesthetic outcomes” AND “aesthetic breast surgery” OR “cosmetic breast surgery” OR “breast surgery” as a “topic” on February 26, 2024. The timespan covered all available years (1953-2024). Inclusion criteria were journal publications from this search strategy, aesthetic-based revision breast surgery publications were also eligible for publication. Exclusion criteria were publications not in English, papers not focused on ABS, animal studies, other surgical procedures, and duplicate publications. The LOE was assessed as per the Oxford Centre for Evidence Based Medicine (OCEBM) system (2011).^12^ Journal-provided LOE was not utilized, and the OCEBM resource was utilized and applied to every publication to ensure consistency in assessment.

**Figure 1.**
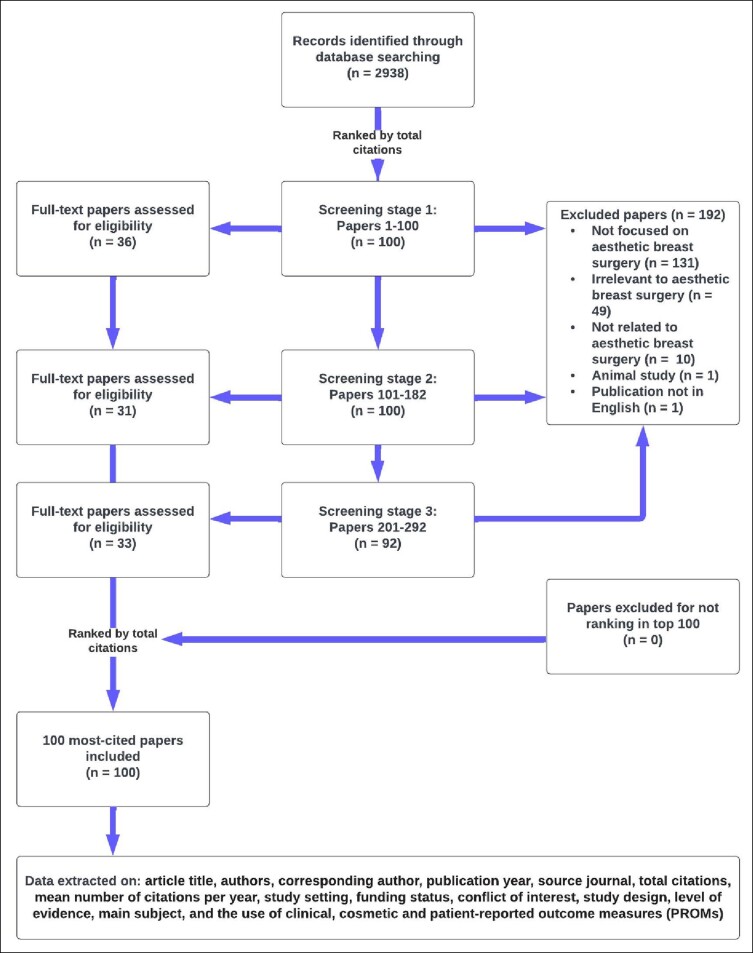
Summary flow chart of methodology. Screening and eligibility allowed the extraction of the 100 most-cited papers.

The search yielded a total of 2938 journal publications, which were subsequently ranked based on the number of times cited. Publications with an equal number of citations were separated using the mean number of citations per year; if the mean number of citations per year were the same, publications were ranked according to the journal impact factor. To ensure that the publications were directly relevant to ABS, 2 reviewers (A.G. and A.C.) independently screened titles and abstracts until 100 journal publications were included. Discrepancies were resolved by consensus discussion with one of the first authors (K.D.), with any remaining inconsistencies being ratified by review of the publication's full text. A total of 292 journal publications were screened to provide the most frequent 100 publications for inclusion. Reasons for the exclusion of the other publications are specified in Figure 1.

Data were extracted from full-text publications in a standardized online computer spreadsheet (Google Sheets: Google LLC, Mountain View, CA). Data extraction included publication title, author list, corresponding author, publication year, source journal, total citations, mean number of citations per year, geographical study setting, funding source, study design, LOE, main subject/content focus, declaration of conflict of interest (CoI) and the use of validated clinical, cosmetic, and patient-reported outcome measures (PROMs), including the BREAST-Q^13^ (Figure 1). For the studies “not focused on ABS,” this was because of the publication topic being breast surgery but not aesthetic focused. “Irrelevant to ABS” were for publications that discussed the breast but not from a surgical perspective. “Not related to ABS” were for publications deemed completely unrelated to the ABS topic focus.

## RESULTS

### Citation Analysis

The 100 most-cited publications on ABS were cited by 11,522 publications.^13-111^ The number of citations accrued per paper ranged from 46 to 1211. Publications were cited with a mean of 115.22 times ± 135.7. The mean number of citations per publication per year ranged from 2.88 to 75.5 (mean 7.25; Table 1).

**Table 1.** The 100 Most-Cited Publications Regarding Aesthetic Breast Surgery

Rank	Author	Last author	Corresponding author	Citations	Mean citations per year
1	Pusic, AL et al	Cano, SJ	Pusic, AL	1211	75.75
2	Coleman, SR and Saboeiro, AP	Saboeiro, AP	Coleman, SR	572	31.78
3	Benelli, L	Benelli, L	Benelli, L	347	9.91
4	Wallace, MS et al	Dobke, MK	Wallace, MS	308	10.62
5	Wise, RJ	Wise, RJ	Wise, RJ	256	3.71
6	Cano, SJ et al	Pusic, AL	Pusic, AL	252	19.38
7	Cohen, WA et al	Pusic, AL	Cohen, WA	245	27.22
8	Ching, S et al	Antony, MM	Thoma, A	245	11.14
9	Klein, SM et al	Greengrass, RA	Klein, SM	230	9.2
10	Pusic, AL et al	Cordeiro, PG	Pusic, AL	227	12.61
11	Pusic, AL et al	Wilkins, EG	Pusic, AL	217	27.13
12	Rietjens, M et al	Petit, JY	De Lorenzi, F	202	11.22
13	Fitoussi, AD et al	Salmon, RJ	Berry, MG	191	12.4
14	Adams, WP et al	Smith, SJ	Adams, WP	175	9.21
15	Olsen, MA et al	Fraser, VJ	Olsen, MA	170	10
16	Missana, MC et al	Balleyguier, C	Missana, MC	170	9.44
17	Asgeirsson, KS et al	Macmillan, RD	Asgeirsson, KS	168	8.4
18	Galdino, GM et al	Manson, P	Galdino, GM	154	6.7
19	Chen, CM et al	Pusic, AL	Pusic, AL	152	10.13
20	Brown, MH et al	Silver, SA	Brown, MH	146	7.3
21	Pusic, AL et al	Cano, SJ	Pusic, AL	136	9.71
22	Kling, RE et al	Rubin, JP	Rubin, JP	133	11.08
23	Mallucci, P and Branford, OA	Branford, OA	Mallucci, P	133	10.23
24	Klassen, AF et al	Cano, SJ	Klassen, AF	132	8.25
25	Penn, J	Penn, J	Penn, J	131	1.87
26	Spear, SL et al	Nahabedian, MY	Spear, SL	125	8.93
27	Codner, MA et al	Nahai, F	Codner, MA	117	8.36
28	Adams, WP et al	Smith, SJ	Adams, WP	113	5.95
29	Grolleau, JL et al	Costagliola, M	Grolleau, JL	111	4.27
30	Munhoz, AM et al	Ferreira, MC	Munhoz, AM	109	5.74
31	Hammond, DC et al	Phillips, CA	Hammond, DC	106	8.15
32	Blount, AL et al	Alfonso, DR	Blount, AL	99	8.25
33	Spear, SL et al	Menon, N	Spear, SL	96	4.57
34	Edsander-Nord, Å et al	Wickman, M	Edsander-Nord, Å	96	4
35	Zuo, KJ et al	Lin, AN	Lin, AN	91	9.1
36	Edgerton, MT et al	Jacobson, WE	Edgerton, MT	90	1.42
37	Metcalfe, KA et al	Pal, T	Metcalfe, KA	90	9
38	Losken, A et al	Carlson, GW	Losken, A	90	6.43
39	Edgerton, MT and McClary, AR	McClary, AR	Edgerton, MT	90	1.34
40	Kakagia, D and Pallua, N	Pallua, N	Kakagia, D	87	7.91
41	Fitzal, F et al	Wild, T	Fitzal, F	86	4.78
42	Tepper, OM et al	Karp, NS	Tepper, OM	85	4.47
43	Cano, SJ et al	Pusic, AL	Pusic, AL	84	7
44	Ueda, S et al	Noguchi, S	Noguchi, S	84	4.94
45	Rohrich, RJ et al	Foster, B	Brown, SA	83	3.95
46	Spear, SL et al	Clemens, MW	Spear, SL	81	4.26
47	Jacobson, JM et al	Spear, SL	Spear, SL	80	6.15
48	Spear, SL et al	Al-Attar, A	Spear, SL	78	6
49	Romundstad, L et al	Stubhaug, A	Romundstad, L	78	4.11
50	Hyakusoku, H et al	Hirakawa, K	Ogawa, R	77	4.81
51	Blondeel, PN et al	Landuyt, KV	Blondeel, PN	76	4.75
52	Barone, M et al	Persichetti, P	Cogliandro, A	72	9
53	Pirro, O et al	Bassetto, F	Mestak, O	72	9
54	Gendy, RK et al	Rainsbury, RM	Gendy, RK	72	3.27
55	Maxwell, GP and Gabriel, A	Gabriel, A	Maxwell, GP	71	4.44
56	Gonzalez, MA et al	Simpson, RL	Glickman, LT	70	5.38
57	Swanson, E	Swanson, E	Swanson, E	69	5.31
58	Hartzell, TL et al	Slavin, SA	Slavin, SA	69	4.6
59	Ganott, MA et al	Costa-Greco, MA	Ganott, MA	68	2.06
60	Pfeiffer, P et al	Hölmich, LR	Pfeiffer, P	67	4.19
61	Groen, J-W, et al	Smit, JM	Mullender, MG	66	7.33
62	Lista, F et al	Ahmad, J	Ahmad, J	66	5.5
63	Macmillan, RD and McCulley, SJ	McCulley, SJ	Macmillan, RD	64	7.11
64	Hester, TR et al	Stokes, L	Hester, TR	64	4.92
65	Somogyi, R and Brown, MH	Brown, MH	Brown, MH	62	6.2
66	Graf, R et al	Biggs, T	Graf, R	62	2.82
67	Kovacs, L et al	Machens, HG	Kovacs, L	61	4.69
68	Eder, M et al	Kovacs, L	Kovacs, L	59	4.54
69	Berry, MG and Stanek, JJ	Stanek, JJ	Berry, MG	58	4.46
70	Hammond, DC et al	Capraro, PA	Hammond, DC	58	2.64
71	Spear, SL et al	Al-Attar, A	Spear, SL	57	4.75
72	Swanson, E	Swanson, E	Swanson, E	57	4.75
73	Eder, M et al	Kovacs, L	Kovacs, L	57	4.07
74	Avsar, D et al	Taşkinalp, O	Avsar, D	57	3.8
75	Caruso, F et al	Castiglione, G	Catanuto, G	57	3.35
76	O’Connell, RL	Rusby, JE	Rusby, JE	56	5.6
77	Caplin, DA	Caplin, DA	Caplin, DA	56	5.09
78	Blondeel, PN et al	Landuyt, KV	Blondeel, PN	56	3.5
79	Tepper, OM et al	Karp, NS	Karp, NS	55	3.67
80	Spear, SL and Pittman, T	Pittman, T	Spear, SL	54	4.91
81	Paik, AM et al	Lee, ES	Paik, AM	54	4.91
82	Moyer, HR et al	Losken, A	Moyer, HR	53	4.08
83	Hermans, BJE et al	van der Hulst, RRWJ	van der Hulst, RRWJ	53	2.65
84	Bames, HO	Bames, HO	Bames, HO	53	0.74
85	Ahmadi, AH et al	Shayani, P	Ahmadi, AH	52	2.65
86	Lesavoy, MA et al	Dickinson, BP	Lesavoy, MA	52	3.47
87	Stevens, WG et al	Stoker, DA	Stevens, WG	52	3.06
88	Rocco, N et al	Nava, MB	Rocco, N	51	5.67
89	Hamdi, M	Hamdi, M	Hamdi, M	51	4.25
90	Sears, ED et al	Chung, KC	Chung, KC	51	2.83
91	Hardwicke, JT et al	Skillman, JM	Hardwicke, JT	50	4.17
92	McIntosh, J and O’Donoghue, JM	O’Donoghue, JM	McIntosh, J	50	3.85
93	Liu, YJ and Thomson, JG	Thompson, JE	Thomson, JG	50	3.57
94	Gupta, V et al	Higdon, KK	Gupta, V	49	6.13
95	Munhoz, AM et al	Gemperli, R	Munhoz, AM	49	4.08
96	Cooter, RD et al	Gardiner, SE	Cooter, RD	48	2.67
97	Anderson, RC et al	Lenderking, WR	Anderson, RC	47	2.53
98	Becker, H and Lind, JG	Lind II, JG	Becker, H	47	3.92
99	Hanemann, MS and Grotting, JC	Grotting, JC	Hanemann, MS	47	3.13
100	Dragu, A et al	Ingianni, G	Dragu, A	46	2.88

The most frequently cited publication (*n* = 1211) was, authored by Pusic et al, and published in 2009. The authors described a novel strategy to measure patient-reported outcomes because of their increasing importance in cosmetic and reconstructive breast surgery, known as the BREAST-Q. This conception allows patients to quantify their satisfaction and promotes patient advocacy.^13^ The publication numbers for corresponding authors were recorded, and the outcomes were compared first. Pusic and Spears both had the largest number of authorships as corresponding authors, at 7 each, with Kovacs at 3, followed by Adams, Berry, Blondeel, Brown, Edgerton, Hammond, Munhoz, and Swanson all tied at 2 (Table 1). Following corresponding authorships being calculated, active publications were counted next, with values for authors being calculated by the frequency of first authorships. Spear was the author with the largest number of publications, with 6 in total. This was followed by Pusic with 4 publications. Lastly, there were 6 authors tied on 2 publications; however, Blondeel, Munhoz, Swanson, and Hammond all additionally had 2 corresponding authorships (Table 1). There is a correlation between senior authors of a publication and being the last author; consequently, last authorships were also calculated, with Pusic having the most (*n* = 4), followed by Cano (*n* = 3) and Al-Attar, Karp, Kovacs, Landuyt, Smith, and Swanson all being tied on 2 (Table 1).

**Figure 2.**
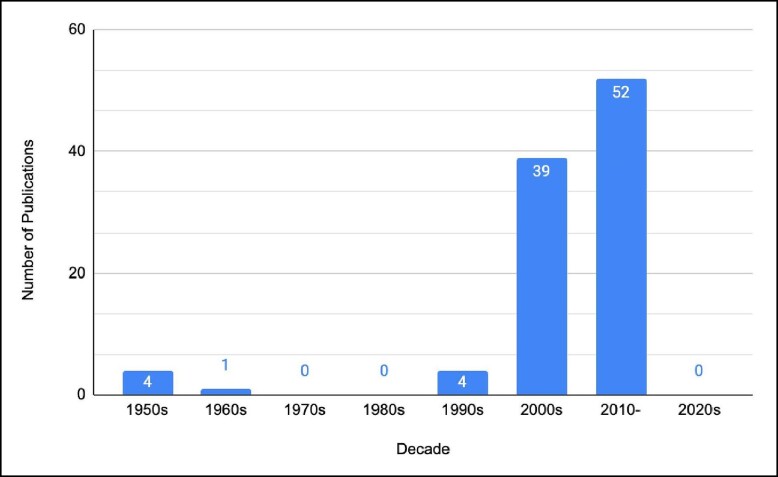
Decade analysis of the 100 most-cited publications in aesthetic breast surgery. These publications increase exponentially as decade progress.

A significant portion of the most frequently cited publications were published in the 2000s and 2010 onwards, coming at 39 and 52 publications, respectively. The most frequently cited publication was published throughout this post 2000s time period (Figure 2). The United States of America was the country with the highest number of publications (*n* = 54), followed by the United Kingdom (*n* = 8) and Canada (*n* = 7; Table 2).

**Table 2.** Country Frequency in the 100 Most-Cited Aesthetic Breast Surgery Publications

Rank	Country	No. of publications
1	United States of America	54
2	United Kingdom	8
3	Canada	7
4	Germany	4
5	France	4
6	Italy	4
7	Brazil	3
8	Belgium	3
9	The Netherlands	2
10	Japan	2
11	Australia	1
12	Austria	1
13	Czech Republic	1
14	Denmark	1
15	Greece	1
16	Norway	1
17	South Africa	1
18	Sweden	1
19	Turkey	1

Nineteen studies formally acknowledged the receipt of funding, a smaller proportion of funding (*n* = 7) was provided by various private entities, such as Allergan (Allergan Aesthetics, an AbbVie Company, Irvine, CA) and Byron Medical (Tucson, AZ). Four publications stated the receival of a grant from a university or university-based faculty leadership. The remaining 15 funding sources included the National Institute of Health, Plastic Surgery Foundation, and various charitable and private corporations. Thirty-two explicitly stated receipt of no funding, the remaining 49 studies did not specify whether they received funding or not from external (governmental, industry, institutional, etc) or internal (departmental, divisional levels of organizations) sources. Additionally, 26 publications disclosed potential CoI with most authors stating that they were consultants or held equity in and for the companies providing products used. A further 27 publications explicitly stated that there were no CoI's to declare and the remaining 47 did not specify whether there was any CoI or not.

### Prevalent Research Themes

The most-cited 100 publications regarding ABS originated from 25 journals. *Plastic and Reconstructive Surgery* contributed the most publications (*n* = 44), followed by *Aesthetic Surgery Journal* (*n* = 12), with *The Breast Journal* next (*n* = 7), then *Journal of Plastic Reconstructive and Aesthetic Surgery* (*n* = 6). The remaining journals contributed 30 publications between them (Table 3). Most of the publications selected for this analysis were from dedicated *Plastic Surgery Journals* such as *Plastic and Reconstructive Surgery* and *Journal of Plastic Reconstructive and Aesthetic Surgery*. Twenty-six publications on the list were published in journals not specifically related to plastic surgery which include *The Breast Journal*, *European Journal of Surgical Oncology*, *Pain, Anaesthesia and Analgesia*, *Annals of Surgical Oncology*, *Archives of Surgery*, *BMC Women's Health*, *British Journal of Surgery*, *Cochrane Database of Systematic Reviews*, *Current Breast Cancer Reports*, *Gland Surgery*, *Journal of Clinical Oncology*, *Radiography*, *Surgery*, *Surgical Innovation*, and *American Journal of Surgery.* Among all the platforms, *British Journal of Surgery* has the highest impact factor (*n* = 9.6).

**Table 3.** Journal Frequency in the 100 Most-Cited Aesthetic Breast Surgery Publications

Rank	Source journal	No. of publications	Impact factor
1	*Plastic and Reconstructive Surgery*	44	3.6
2	*Aesthetic Surgery Journal*	12	2.9
3	*The Breast Journal*	7	2.1
4	*Journal of Plastic, Reconstructive and Aesthetic Surgery*	6	2.7
5	*Aesthetic Plastic Surgery*	5	2.4
6	*Annals of Plastic Surgery*	5	1.5
7	*European Journal of Surgical Oncology*	4	3.8
8	*Pain*	2	7.4
9	*American Journal of Surgery*	1	3
10	*Anaesthesia and Analgesia*	1	5.7
11	*Annals of Surgical Oncology*	1	3.7
12	*Archives of Surgery*	1	3.45
13	*BMC Women's Health*	1	2.5
14	*British Journal of Surgery*	1	9.6
15	*Clinics in Plastic Surgery*	1	2.3
16	*Cochrane Database of Systematic Reviews*	1	8.4
17	*Current Breast Cancer Reports*	1	0.8
18	*Gland Surgery*	1	1.8
19	*Journal of Clinical Oncology*	1	45.3
20	*Plastic and Reconstructive Surgery—Global Open*	1	1.5
21	*Radiography*	1	2.6
22	*Surgery*	1	3.8
23	*Surgical Innovation*	1	1.5

**Figure 3.**
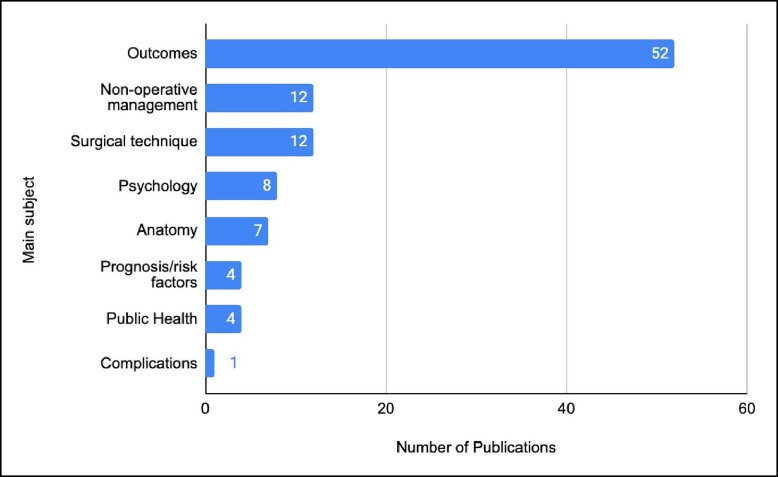
Main subjects of the 100 most-cited publications on aesthetic breast surgery. Outcomes as a main subject have more publications than that of the other main subject categories combined.

The main subject in a majority of the publications was “outcomes” (*n* = 52). Followed by “nonoperative management” and “surgical technique” tied together (*n* = 12). Publications regarding surgical technique mostly discuss autologous fat transfer for breast augmentation. Whereas publications with outcomes as the main subject deliberated aesthetic outcomes for breast surgery, many of which utilized the BREAST-Q tool (Figure 3).^13^ Most of these studies have been conducted in the last 2 decades corresponding to an increase in demand for these procedures due to various aesthetic, social, and psychological reasons. However, the top 3 cited articles were conducted prior to 2010.^13,14^ The field of ABS was also calculated within the top 100 publications. Many studies discussed multiple fields, for example, 1 cohort undergoing augmentation others mastopexy, these were counted as individual points, thus the total value exceeding 100. Augmentation was the most the populous field of ABS (*n* = 55); the remaining results are summarized in Table 4.

**Table 4.** Field of Aesthetic Breast Surgery in the 100 Most-Cited Aesthetic Breast Surgery Publications

Field of ABS	Frequency of association within studies
Augmentation	55
Reduction mammaplasty	28
Augmentation mastopexy	11
Augmentation mammaplasty	10
Mastopexy	8
Symmetry	3
Mastopexy reduction	1

ABS, aesthetic breast surgery.

### Methodological Quality

Almost half of the studies are cohort studies with 25 following a retrospective model while 19 being prospective in nature. The analysis also involves multiple review papers and case series (*n* = 21). Only 1 randomized controlled trial (RCT) is included in this study.

**Figure 4.**
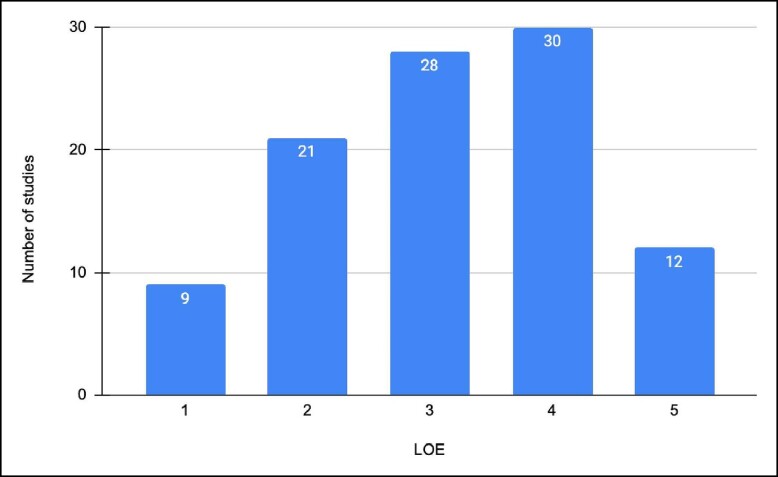
The levels of evidence for the 100 most-cited aesthetic breast surgery publications. Most papers were levels of evidence 3, with more of the remaining papers being a lower level of evidence than higher.

**Figure 5.**
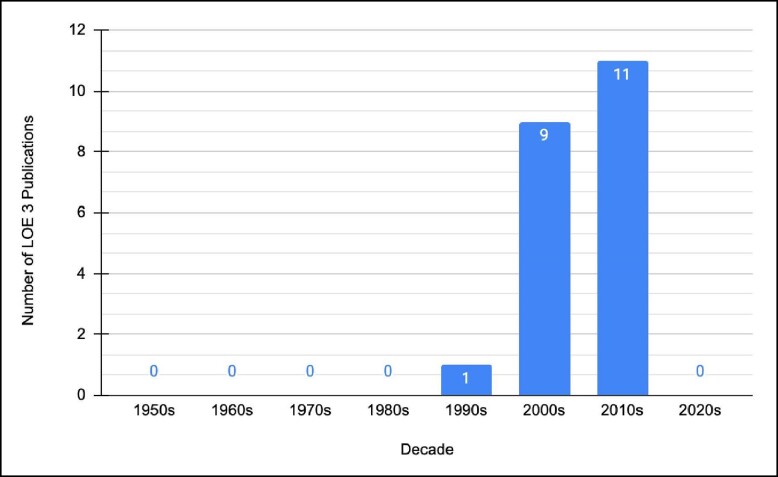
Number of levels of evidence 3 publications vs decade within the 100 most-cited aesthetic breast surgery publications. The vast majority of these papers were published in more recent decades. LOE, level of evidence.

Almost a third of the publications on the list were assessed to be OCEBM LOE 4 (*n* = 30); this was represented mostly by case series studies. Twelve publications achieved LOE 5, whereas 28 publications achieved LOE 3. An additional 21 publications achieved LOE 2 and lastly 9 publications achieved LOE 1 (Figure 4). LOE 1 corresponds to: systematic review of (mostly) RCTs or individual RCTs. LOE 2 corresponds to: poorly designed RCTs, prospective cohort studies, or systematic reviews of cohort studies. LOE 3 corresponds to: retrospective cohort studies and case–control studies. LOE 4 corresponds to: case series. LOE 5 corresponds to: case reports and expert opinions. Upon observation of decade analysis, research output, in terms of the number of publications, greatly increased with each decade passing, until the 2020s (1980s: *n* = 2; 1990s: *n* = 4; 2010: *n* = 52; 2020s: *n* = 0). Additionally, the proportion of publications (0:1:9:17:0 mapped to 1980s:1990s:2000s:2010s:2020s) with LOE 3 increased consecutively with each decade post 1990s (Figure 5). Study designs of the 100 most-cited research are presented in Table 5.

**Table 5.** Study Design Frequency in the 100 Most-Cited Aesthetic Breast Surgery Publications

Rank	Study design	No. of publications
1	Retrospective cohort study	25
2	Case series	21
3	Prospective cohort study	19
4	Expert opinion	12
5	Systematic review	8
6	Narrative review	5
7	Cross-sectional study	3
8	Literature review	3
9	Experimental study	1
10	Randomized trial	1
11	Observational study	1
12	Systematic review and meta-analysis	1

Clinical outcomes were reported in 46 of the top 100 most highly cited publications. Outcome measures were categorized in studies. Twenty-nine publications incorporated PROMs, namely patient satisfaction and the BREAST-Q questionnaires. Fifty-three publications incorporated cosmetic outcome measures, including preoperative and postoperative photography, breast measurement, and breast symmetry index.^52^

## DISCUSSION

ABSs are popular plastic surgery procedures that are continuously evolving.^5,6^ This is the first bibliometric analysis to the best of our knowledge that evaluates the 100 most-cited articles on ABS involving breast augmentation, mastopexy, and reduction. The most-cited article in the analysis was authored by Pusic et al. This focused on the development of the BREAST-Q questionnaire. BREAST-Q for ABS can be used to provide quantifiable data to determine patient satisfaction for cosmetic-related breast procedures.^13^

There is a need for further investigation with a larger patient pool and for the development of additional PROMs. Overall PROMs were lacking across the 100 most-cited studies, and when they were implemented, the BREAST-Q was the favored PROM of choice. The study pertaining to the conception of the BREAST-Q represents a landmark paper in the field of ABS and PROMs, which has shaped the trajectory of future research and further nurtured the doctor–patient relationship.^13^ It should be noted that BREAST-Q was developed in 2009, and this bibliometric analysis captures studies both before and after the development of this PROM. Forty studies were published prior to 2009, and therefore were unable to utilize BREAST-Q. Only 9 out of the 60 remaining studies used BREAST-Q, which can be extrapolated to a 15% utilization rate.

The second most-cited article was “Fat grafting to the breast revisited: safety and efficacy” authored by Coleman and Saboeiro.^14^ This retrospective cohort study conducted in the United States of America focused on fat grafting (lipo-modeling/lipo-filling) of breasts, its safety, efficacy, and cosmetic effects. The study involved structural fat grafting by harvesting fat cells, centrifuging them, and injecting them directly into one or both breasts. All patients who returned for follow-up being pleased with the increased size and improved contour. The study raised concerns about the potential necrosis of transferred fat tissue, but long-term radiologic follow-up showed that the necrotizing fat tissue could be easily distinguished from breast malignancies on imaging.^14^ A major drawback is the potential for donor-site deformities to occur, and the maximum volume gain from 1 session of fat grafting constituted only 1 cup size. Additionally, in structural fat grafting, fatty tissue is evenly distributed within the breast. This widespread integration of fatty tissue does not result in the same noticeable visual volume increased compared with artificial implants. However, volume MRI studies can provide a more accurate quantification of the survival of a specific volume of fat placed into the breast.^14,113,114^

Both studies discussed above hold great relevance to ABS. The first study introduced an innovative method of assessing patient satisfaction and health-related quality of life within aesthetic and non-ABS.^13^ The second study addresses concerns about the safety and efficacy fat grafting into breasts. Given specific techniques are utilized, satisfactory, long-lasting, and natural improvements in the size and shape of the breast can be achieved.^14^

The third most-cited study in the list, “A new periareolar mammaplasty—the ‘round block’ technique” authored by Benelli and published in *Aesthetic Plastic Surgery* was cited 347 times.^112^ This study design was an expert opinion that focused on the discussion of the “round block” technique, which supports the mammary cone and aims to obtain good breast shape within the areola, free from tension that would cause postoperative enlargement. The study concluded that this technique could be utilized in various aspects of breast surgery: in cases of ptosis or hypertrophy. The use of the round block technique presents with 2 advantages, leaving an inconspicuous scar and avoidance of depression around the area of excision.^112^

A consensus on the definition of attractive breasts remains elusive, as attractiveness is inherently influenced by sociocultural factors, as proposed by the tripartite influence model.^1,6^

In a 1997 publication, Westreich attempted to establish a standardized preoperative evaluation protocol for achieving “aesthetically pleasing” breasts.^115^ He defined the ideal breast as a “nonptotic in which no common aesthetic procedure would be considered appropriate to enhance the breast form.”^6^ Westreich's study involved 50 females with perceived perfect breasts. The study identified statistically significant correlations between certain parameters of breast, torso shape, and breast volume. However, Westreich’s work built upon the contributions of previous authors, such as Penn and Smith.^37,116^ He acknowledged discrepancies in their respective protocols, particularly regarding the ideal nipple plane and inframammary crease length.^6^ The complexity of objectively classifying the breast as an organ is apparent, with variations in subjective beauty definition.^6^ Further studies have been conducted since to delineate the correct measurements and anatomical ratios for ABS.^3,117^ The 23rd most-cited study within this analysis was the landmark Mallucci and Branford paper, which coined the “45:55” ratio.^35^ This observational study aimed to identify objective indices for the ideal aesthetic breast. The natural breasts of 100 topless models were analyzed photographically to determine the ideal attractive breast. Analysis revealed a ratio of 45:55, representative of upper pole:lower pole, a straight-line upper pole slope, upward pointing nipple and lower pole convexity to be the ideal standard, and thus, the future aim for the breast augmentation-based procedures. This study established objective guidelines that ultimately serve as a potential template for the design of aesthetic breasts, thus providing predictable, repeatable outcomes that the patient can be satisfied with.^35^

A survey conducted by Bekisz et al in 2023, collected 1021 responses from a diverse population.^118^ The primary objective of the study was to evaluate the aesthetic and anatomical characteristics valued by patients seeking ABS. According to this study, females generally preferred “moderate”-sized breasts with a greater upper pole fullness to lower pole ratio.^35^ We believe there should be further research delving into what patients consider aesthetically pleasing breasts, rather than on the perceptions of surgeons.^6^ Given the variable outcomes of ABS, it is essential to develop a strong doctor–patient relationship during the consultation, understand the expectations of the patient, and explain outcomes in a way that aligns with realistic patient expectations. We encourage further large-scale studies focusing on patients’ definition of aesthetically pleasing breasts.

The studies in our bibliometric analysis mainly involved observational studies. These types of studies can have many biases like observational bias, pygmalion bias (what people expect from others can influence an outcome, eg, a superiors’ positive prediction of one's performance can improve outcome, and vice versa), or measurement biases. Many unaccounted biases can also be part of the overall picture. Often recall bias can distort the result especially in the scenario where patients’ satisfaction is being assessed as an outcome. Overall, most of the studies in the analysis exhibit a low LOE, lack proper follow-up, and feature small sample sizes.

These factors raise concerns about the authenticity of the results, emphasizing the need to address them by conducting these methods over a larger population sample and following them over a more extended period. Conducting RCTs should be a priority because observational studies often have inherent biases that make it difficult to establish causation with confidence. Nevertheless, it should be acknowledged that RCTs in the ABS patient population can be difficult to undertake. There is a possibility that relevant RCTs were present within our search but did not appear within the top 100 studies. This bibliometric analysis highlights the deficiency in utilization of validated PROMs in ABS. Future research should aim to incorporate this to better understand what classifies a good outcome, as well as help develop objective, patient-centered definitions of what an aesthetic breast is.

The limitations of this study include those associated with the bibliometric analysis methodology. Citation choice is open to partiality, such as citation bias, distortion, amplification, and invention, which may collectively result in the unfounded authority of certain publications. Although constructing a citation network to investigate this was beyond the scope of this review, we analyzed only the most-cited research, thus minimizing the effect of such bias.^119^ Additionally, there is an assumption that citations equate to the utility of a study; however, it could be stated that “views” or “downloads” would be a better metric of how a publication's access has been reached, and this has been disputed in recent years. With the usage of empirical evidence, the bibliometric methodology is preferable.^120^

Encouraging further research on the topic of ABS, the study suggests that large trials can provide a standardized procedure for performing ABS. The study aims to provide a general outline to future researchers about the current trends in ABS research and hopes to contribute to the improvement of these trends.

## CONCLUSIONS

This extensive bibliometric analysis comprehensively examines the top 100 most highly cited publications regarding ABS and shows the evolution and trends in the field over the past 8 decades. Emerging research areas within this field include a multitude of refinements in safety and surgical technique to optimize for aesthetic outcomes and mitigate capsular contraction. Improvements in the quality of ABS literature must be sought by active prioritization of the publication of methodologically robust studies with higher OCEBM LOE, such as well-designed RCTs or multicenter studies. Furthermore, the adoption of validated PROMs designed for ABS is centrally important for aligning patient satisfaction with clinical outcomes and providing high-quality evidence-based patient care.
